# Analysis of Phase Mismatch for Mercurous Bromide-Based Non-Collinear AOTF Design in Spectral Imaging Applications

**DOI:** 10.3390/ma17071703

**Published:** 2024-04-08

**Authors:** Huijie Zhao, Chi Cheng, Qi Guo, Rui Ma, Yutian Yang

**Affiliations:** 1 School of Instrumentation and Opto-Electronic Engineering, Beihang University, Beijing 100191, China; chengchi93@163.com (C.C.); marui1108@buaa.edu.cn (R.M.);; 2 Institute of Artificial Intelligence, Beihang University, Beijing 100191, China; 3 Aerospace Optical-Microwave Integrated Precision Intelligent Sensing, Key Laboratory of Ministry of Industry and Information Technology, Beihang University, Beijing 100191, China; 4 Qingdao Research Institute of Beihang University, Qingdao 266104, China

**Keywords:** AOTF, AOTF design, mercurous bromide

## Abstract

The spectral and spatial characteristics of Acousto-Optic Tunable Filters (AOTFs), such as a tuning curve, spectral resolution, angular aperture, and diffraction efficiency, are determined by the device’s acousto-optic crystal configuration and piezoelectric transducer. For high-throughput spectral imaging applications, it is essential to enlarge the spectral bandwidth and angular aperture during the design phase of AOTFs. Thus, phase mismatch due to incident angle or wavelength was studied analytically using phase diagrams in this paper. Additionally, a performance parameter analysis model was established based on the use of mercurous bromide crystals for large angular aperture AOTF device design, and the impact of crystal and transducer design parameters on the spectral bandwidth and angular aperture was evaluated. This also experimentally validates the diffraction capability of AOTFs made from mercurous bromide crystal, which possess a broad spectral transmission ability ranging from visible to long-wave infrared.

## 1. Introduction

Spectral imaging systems based on an Acousto-Optic Tunable Filter (AOTF) possess numerous advantages, including rapid wavelength switching, frame-capture imaging, complete electronic control, and all-solid-state components [1,2]. These systems are capable of acquiring the spatial, radiometric, and spectral information of objects, and have been widely studied in space exploration [3,4,5], hyper-spectral imaging [6], and stereoscopic imaging [7]. The AOTF is a spectral dispersive device that operates based on the elasto-optical effect, which refers to the periodic modulation of the refractive index caused by acoustic waves traveling through an optically transparent medium [8]. The incident light waves of specific wavelengths propagating through the medium are diffracted to a certain direction. Thus, AOTF can separate the energies of different light wavelengths from the incident light by switching the frequency of the driving radio-frequency (RF) electrical signal. AOTF comes in various layout structures, which can be categorized according to the direction of the acoustic waves in relation to the incident light waves as collinear, non-collinear, and quasi-collinear types. Compared with collinear AOTFs, the diffracted and transmitted light in non-collinear AOTFs naturally separates in space, which is beneficial for spectral imaging systems that utilize diffracted light [9]. Spectral imaging systems require a high ability to collect energy in order to enhance signal quality. As one of the core components of the spectral imaging system, the AOTF directly affects many critical performances of the spectral imaging system, such as the spectral resolution, field of view, and sensitivity. Among these, the optical throughput of the AOTF is the main limiting factor of the system’s energy-collecting ability and signal quality, especially in the infrared band. The optical throughput of the AOTF device is directly determined by characteristics such as spectral resolution, the angular aperture, and the product of transmission and diffraction efficiency.

The angular aperture of the non-collinear AOTFs developed in the early stages was very small (about 1 mrad) [10]. Chang introduced the Parallel Tangent Principle (PTP) to achieve a wide angular aperture [10]. The theoretical formula for the non-collinear AOTF proposed by Chang laid the foundation for AOTF and system design, which is applicable across various spectrums ranging from visible [11] to long-wave infrared [12], from conventional acousto-optic (AO) crystals [13] to new types of AO crystals [14], and from single-channel [15] to multi-channel types [16]. In addition, quasi-collinear devices are also a hot topic of recent research, with the advantage of achieving ultra-high spectral resolution and diffraction efficiency through very long acousto-optic interaction lengths [17].

The types of medium used for acousto-optic interactions are quite diverse [18]. Typically, the optically transparent medium used for the interaction between acoustic waves and optical waves in AOTF devices discussed in this paper is an anisotropic AO crystal, and the performance of the AO crystal is crucial to the device’s characteristics. Currently, the commercially mature AO crystal is tellurium dioxide (TeO_2_), which has high transmittance in the 0.35 μm–5 μm range and excellent acousto-optic properties [19]. However, the AOTF field is still actively seeking better-performing crystals, especially those that can transmit in the mid- to long-wave infrared spectrum. The mercurous bromide (Hg_2_Br_2_) crystal proved to be outstanding at producing broadband spectral devices due to its advantages of a broad transparent range with high transmittance from visible to long-wave infrared bands (0.42–30 μm) [20]; high acousto-optic figure of merit of 2600 (×1.5 × 10^−18^ s^3^/g), which characterizes the diffraction efficiency of acousto-optic devices, while the commonly used TeO_2_ has an acousto-optic figure of merit of 800 (×1.5 × 10^−18^ s^3^/g) [21,22]; and large birefringence, which characterizes the angular aperture of acousto-optic devices [23,24]. The challenge in Hg_2_Br_2_ crystal AOTF research is the availability of high-quality AOTF, resulting from difficulties in the growth of high-quality, large-size crystals and the instabilities in the bonding of piezoelectric transducers. Currently, promising preliminary results have been achieved by Hg_2_Br_2_ crystal devices for long-wave infrared imaging [25]. Nevertheless, since the characteristics of the Hg_2_Br_2_ crystal are different from those of TeO_2_, which may lead to different design methods being used for the development of TeO_2_ crystal AOTFs, it is essential to research the design of Hg_2_Br_2_-based AOTFs for various application requirements.

This study theoretically investigated the design of non-collinear AOTF and fabricated a prototype with Hg_2_Br_2_ crystal. The phase-matching geometries and energy decline caused by phase mismatches were explored. The impact of the AO crystal’s cut parameters and the dimensions of the piezoelectric transducers on the device’s driving frequency, monochromatic angular aperture, and spectral bandwidth were analyzed, providing guidance for the design of AOTF devices with mercurous bromide crystals.

## 2. Methods

**Figure 1:**
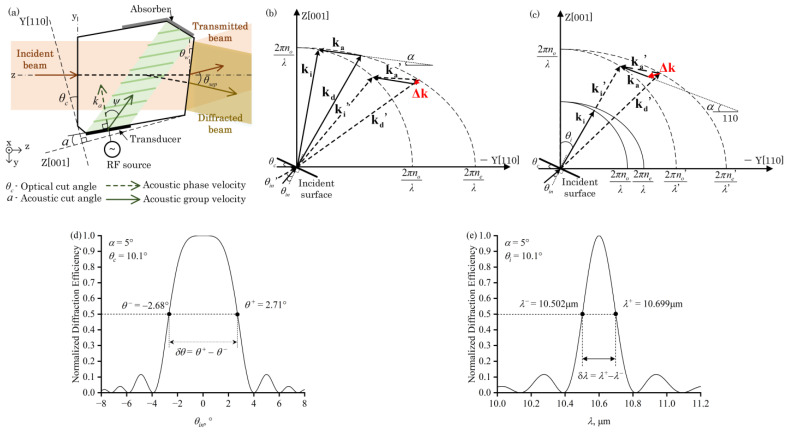
(**a**) Configuration of non-collinear AOTF. The crystal coordinate system’s *y*-axis and *z*-axis are aligned with the acousto-optic crystal’s crystallographic axes, [110] and [001], respectively, and the system coordinate’s *z*-axis is aligned with the normal to the incident surface. *θ_c_* is the optical cut angle, *α* is the acoustic cut angle, and *θ_w_* is the exit wedge angle. (**b**) Diagram of phase mismatch caused by external incident angle. Solid lines represent phase-matching and dashed lines represent phase-mismatching. (**c**) Diagram of phase mismatch caused by incident optical wavelength. (**d**) Normalized diffraction efficiency varies with the external incident angle. (**e**) Normalized diffraction efficiency varies with the incident optical wavelength.

The AOTF operates based on the principle of anisotropic Bragg acousto-optic interaction. As shown in Figure 1a, the incident light illuminates the incident surface of the AO crystal. After the refraction on the incident surface, the acousto-optic interaction occurs within the AO crystal when the incident light satisfies the phase-matching condition with the acoustic wave, filtering out diffracted light of a specific central optical wavelength. Subsequently, the diffracted light and non-diffracted light separately undergo refraction at the exit surface. The acousto-optic interaction process is represented with wave vectors, as shown by the solid lines in Figure 1b,c, when the acoustic wave vector is **k_a_** and the wave vectors of the incident and diffracted light, **k_i_** and **k_d_**, form a closed triangle, the phase matching condition is met, which is represented as follows:

(1) $$k_{i}\pm k_{a}=k_{d}$$

The direction of the incident light wave vector **k_i_** is normal to the incident wave, with a magnitude of *k_i_* = 2π*n_i_*/*λ*. The diffracted light wave vector **k_d_** is in the direction normal to the diffracted wave, with a magnitude of *k_d_* = 2π*n_d_*/*λ*, where *λ* is the wavelength of the light wave, and *n_i_* and *n_d_* are the optical refractive indices of the incident and diffracted light, respectively. The acoustic wave vector **k_a_** forms an angle α with the [001] crystal axis, with a magnitude of *k_a_* = 2π*f_a_*/*v_a_*, where *f_a_* is the frequency of the acoustic wave, which is also the frequency of the RF electrical signal’s input to the piezoelectric transducer and *v_a_* is the velocity of the acoustic wave. In AO crystals, the refractive index is determined by the polarization and the direction of the optical wave. The polarization of the incident light and the diffracted light in AO crystals are orthogonal to each other. The light with ordinary polarization is represented as (o). The light with extraordinary polarization is represented as (e). When the incident light is (o), the diffracted light is (e). Therefore, *n_i_* and *n_d_* are the refractive indices for (o) and (e), respectively. Due to the anisotropy of uniaxial crystals, the extraordinary refractive index varies with the angle *θ_i_* between the light wave and the [001] crystal axis, whereas the ordinary refractive index remains constant. The refractive indices are represented as follows [22]:

(2) $$\begin{array}{l} n_{i}=n_{o}\\ n_{d}={n_{e}}^{\prime }\left(\theta_{i}\right)=\sqrt{\frac{{n_{o}}^{2}{n_{e}}^{2}}{{n_{o}}^{2}\sin^{2}\theta_{i}+{n_{e}}^{2}\cos^{2}\theta_{i}}} \end{array}$$

Here, *n_o_* and *n_e_* are the principal refractive indices of the AO crystal. The crystal is also anisotropic to acoustic waves, so the velocity of the acoustic wave varies with the direction of the acoustic wave vector, which is determined by the acoustic cut angle *α*, given by [22]:

(3) $$\upsilon_{a}=\sqrt{{V_{110}}^{2}\cos^{2}\alpha +{V_{001}}^{2}\sin^{2}\alpha }$$

where V_110_ and V_001_ are the velocities of the shear slow wave along the [110] and [001] crystal axis. Moreover, there is a deviation between the direction of the acoustic wave vector and the direction of the acoustic group velocity, as shown in Figure 1a. The walk off angle *ψ* between the group velocity and phase velocity of the acoustic wave is related to the acoustic angle *α,* as follows:

(4) $$\psi \left(\alpha \right)=\arctan\left(\frac{{V_{001}}^{2}}{{V_{110}}^{2}}\tan\alpha \right)-\alpha$$

The direction of the optical incidence and acoustic wave in the AOTF device is primarily controlled by two facet cuts of the AO crystal (Figure 1a). One is the optical cut angle *θ_c_* between the incident surface of the AO crystal and the [110] crystal axis, which is represented as the Y axis in the crystal coordinate system. The other is the acoustic cut angle *α* between the transducer facet of the AO crystal and the [001] crystal axis, which is represented as the Z axis in the crystal coordinate system. Additionally, the external separation angle *θ_sep_*, which is the angle between the transmitted and diffracted light in air, is related to the wedge angle *θ_w_* of the exit surface. The *θ_w_* is also fine-tuned to compensate for the chromatic aberration of the diffracted light [24], or adjusted according to the design requirements of multiplex polarization designs [26].

When an AOTF device operates in a practical spectral imaging system, it always lets the principal ray of the on-axis image point satisfy the phase-matching condition with the acoustic waves. Phase mismatch occurs for other forms of light due to a deviation in the incident angle, leading to a reduction in diffraction efficiency, as shown in Figure 1b. The angular aperture is defined as the incident angle deviation corresponding to half of the peak efficiency (Figure 1d). Therefore, the angular aperture is one of the crucial factors limiting the optical throughput that the AOTF-based spectral imaging system can receive. In addition to the diffraction efficiency reduction caused by phase-matching, another factor limiting the angular aperture of AOTF-based spectral imaging system is the separation angle between diffracted and transmitted light. The smaller value obtained due to the two factors should be chosen for the angular aperture [27]. Thus, designing an AOTF device with a large angular aperture and wide separation angle is the foundation for achieving high-throughput spectral imaging.

The spectral bandwidth of the diffracted light is caused by the phase mismatch due to wavelength deviation, as shown in Figure 1c. For certain applications requiring high spectral resolution, for instance, harmful gas detection [14], the design of the AOTF device should minimize the spectral bandwidth. However, for the optical high-throughput purpose, we need to find a reasonable tradeoff between the spectral resolution and optical throughput.

The wave vector mismatch **Δk** is introduced to represent the phase mismatch in the acousto-optic interaction, as follows:

(5) $$k_{i}\pm k_{a}+\Delta k=k_{d}$$

Here, the wave vector mismatch **Δk** is directed perpendicularly to the group velocity of the acoustic wave [11]. The decrease in the diffraction efficiency caused by the phase mismatch can be represented as follows:

(6) $$\eta (\alpha ,\theta_{i},L,p)=u^{2}\frac{\sin^{2}\sqrt{u^{2}+{\left(\frac{\Delta kL_{AO}}{2}\right)}^{2}}}{u^{2}+{\left(\frac{\Delta kL_{AO}}{2}\right)}^{2}}$$

Here, *L_AO_* is the acousto-optic interaction length, which is proportional to the length of the piezoelectric transducer, *L*. *u*, which is represented as *u =* [π*L_AO_*(*M_2_p*/2)^1/2^]*/λ*, is a coupling coefficient related to the acousto-optic figure of merit of the AO crystal, *M_2_*, the ultrasonic power density, *p*, and the length of the piezoelectric transducer.

When the external incident angles, *θ*^+^ and *θ*^−^, cause the value of Δ*kL_AO_*/2 to satisfy the condition that reduces *η* to half of *η_p_*, the monochromatic angular aperture is calculated as δ*θ* = *θ*^+^ − *θ*^−^ (Figure 1d). When the increased and decreased wavelengths, λ^+^ and λ^−^, cause the value of Δ*kL_AO_*/2 to satisfy the condition that reduces the diffraction efficiency *η* to half of its peak value, *η_p_*, the spectral bandwidth can be calculated through *δλ* = *λ*^+^ − *λ*^−^ as the full width at half of the maximum (FWHM), as shown in Figure 1e.

**Figure 2:**
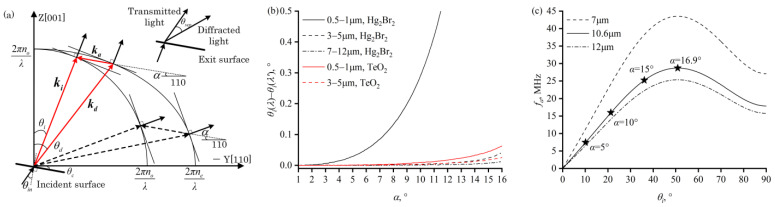
(**a**) Wave vector schematic diagram of Parallel Tangent Principle (PTP) for non-collinear AOTF, where the incident light and the diffracted light have mutually parallel energy velocities within the crystal. For a fixed acoustic angle α, two incident light wave vectors satisfy the PTP, represented by a red solid line and black dashed line. (**b**) The variations in the PTP incident angle cross the 0.5–1 μm, 3–5 μm, and 7–12 μm wavelength range of Hg_2_Br_2_ and TeO_2_ crystals with different acoustic angles. (**c**) The acoustic frequency required for phase-matching in the Hg_2_Br_2_ crystal with different incident angles. The dashed line and the dash-dotted line represent the acoustic frequencies corresponding to the 7 μm and 12 μm optical wavelengths, respectively. The star and the corresponding label indicate the value of the acoustic angle that satisfies the PTP.

In the design of the AOTF-based spectral imaging system, it is common to make the optical cut angle and the acoustic cut angle of the AO crystal follow the PTP [16,28]. This principle stipulates that the tangent of the incident wave vector spherical or ellipsoidal surface at the endpoint of the incident wave vector **k_i_** is parallel to the tangent at the endpoint of the diffracted wave vector **k_d_** (Figure 2a).

When this condition is met, the influence of changes in the angle of light incidence on acousto-optic diffraction is minimized, manifesting as follows. (1) The diffraction efficiency of monochromatic light varies minimally with the incident angle, *θ_i_*, represented by d*η*/d*θ_i_* = 0. Therefore, cutting the incident surface and the transducer surface of the AO crystal according to the PTP can enable the device to obtain a larger angular aperture for monochromatic light. (2) When achromatic light is incident while the acoustic wave vector’s frequency and direction remain constant, and the PTP is satisfied, the central wavelength of the diffracted light changes minimally with the incident angle, *θ_i_*, denoted as d*λ*/d*θ_i_* = 0. Hence, the central wavelength drift of an AOTF that meets the PTP condition is small, which is advantageous for specific imaging optical systems. Additionally, when the PTP is satisfied, the required driving ultrasonic frequency is relatively low.

The PTP forms a constraint between the incident angle, *θ_i_*, and the acoustic angle, *α*. For a specific acoustic angle and a specific light wavelength, only one or two phase-matching combinations satisfy PTP, as shown in Figure 2a. Since the separation angle of the phase-matching combination with a larger incident angle (dashed line) is smaller [29], and an incidence angle close to 90° would complicate the manufacturing of the device, the smaller incident angle and its corresponding acoustic angle (solid line) are selected as the optical cut angle for the incident surface and the acoustic cut angle for the transducer surface of the AO crystal.

When the acoustic angle *α* is fixed, the incident angle, *θ_i_*, that satisfies the PTP changes with wavelength. Hence, for devices with a wide operating wavelength range, the light normally entering the incident surface satisfies the PTP only for a specific wavelength. As shown in Figure 2b, compared to the medium- and long-wave infrared bands, the PTP incident angle is more sensitive to wavelength variations in the visible to near-infrared short-wave spectral bands. Taking an acoustic angle of 5° as an example, the variation of PTP incident angle for Hg_2_Br_2_ crystal in the 0.5–1 μm wavelength range is 0.029°, while, in the 3–5 μm wavelength range, the variation in the PTP incident angle is 0.001°. In the short-wave band, the PTP incident angle of the Hg_2_Br_2_ crystal is more sensitive to wavelength variations than that of TeO_2_. For instance, with an acoustic angle of 5°, the variation in the PTP incident angle for TeO_2_ crystal in the 0.5–1 μm wavelength range is 0.001°, which is much less than that of Hg_2_Br_2_ crystal. As the acoustic angle increases, the PTP incident angle becomes more sensitive to wavelength variations. In the 0.5–1 μm wavelength range, the variation in the PTP incident angle for Hg_2_Br_2_ crystal at an acoustic angle of 5° is 0.029°, and at an acoustic angle of 10°, it is 0.291°; in the 7–12 μm wavelength range, the variation in the PTP incident angle for Hg_2_Br_2_ crystal at an acoustic angle of 10° is 0.001°, and at an acoustic angle of 15°, it is 0.007°.

Based on the phase-matching geometry, a correspondence between the driving frequency and the central wavelength of the diffracted light can be derived, which is known as the tuning curve [30], as follows:

(7) $$f_{a}(\lambda )=\frac{v_{a}}{\lambda }\sqrt{{n_{i}}^{2}+{n_{d}}^{2}-2n_{i}n_{d}\cos(\theta_{d}-\theta_{i})}$$

The required drive frequency for diffraction is inversely proportional to the diffracted light wavelength and is related to the acoustic angle. When the incident angle, *θ_i_*, and the acoustic angle, *α,* satisfy the PTP, the phase-matching acoustic frequency required for the long-wave infrared wavelength range in the Hg_2_Br_2_ crystal is shown in Figure 2c. The dashed and dot-dashed lines represent the acoustic frequencies corresponding to light wavelengths of 7 μm and 12 μm, respectively. Since the acoustic frequency decreases monotonically with the increase in light wavelength, the dashed and dot-dashed lines represent the upper and lower limits of the driving frequencies required for the long-wave infrared band, respectively. The stars and the corresponding tags indicate the acoustic angles that satisfy the PTP for the incident angle. The greater the acoustic angle, the higher the required driving frequency for the same wavelength, and the broader the frequency band range required for the transducer within the same working wavelength range.

## 3. Results and Discussion

The spectral bandwidth, δ*λ*, is related to the acousto-optic interaction length, *L_AO_*. The *L_AO_* is primarily determined by the length of the piezoelectric transducer’s top electrode, *L*. As the light wavelength increases, the spectral bandwidth δ*λ* becomes wider; as the acoustic angle *α* increases, the δ*λ* narrows; as the length of the AOTF’s piezoelectric transducer top electrode *L* increases, the δ*λ* narrows.

The spectral bandwidth of the diffracted light is calculated at a central wavelength of 10.6 μm under different combinations of AOTF design parameters, where the incident angle and the acoustic angle satisfy the PTP. The acoustic angle, *α*, has a significant impact on the spectral bandwidth, δ*λ*. With *L* = 15 mm, to achieve a spectral bandwidth of less than 100 nm, the acoustic angle needs to satisfy α < 6.8°. When *L* = 10 mm, it is only necessary for the acoustic angle to satisfy α < 8.2° to obtain a spectral bandwidth of less than 100 nm.

**Figure 3:**
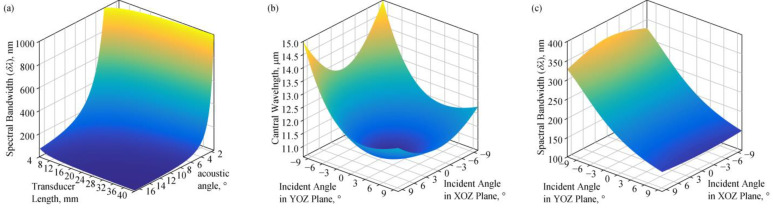
(**a**) The spectral bandwidth of the diffraction light under different acoustic angles, *α*, and incident angles, *θ_i_*, which satisfy the PTP. The central wavelength of diffracted light is 10.6 μm. (**b**) The central wavelength of the diffracted light obtained from light incident at different angles when *α* = 5°, and the optical cut angle, *θ_c_*, is 10.1°. The central wavelength of the diffracted light obtained from a normal incidence on AOTF is 10.6 μm. (**c**) The spectral bandwidth of the diffracted light from light incident at different angles with *α* = 5°, *θ_c_* = 10.1°, *L* = 15 mm.

For the fixed acoustic angle and frequency, diffracted lights have different central wavelengths, caused by polychromatic light entering from various directions [31], and there are also differences in the spectral bandwidth of the diffracted light. As shown in Figure 3b,c, when the acoustic angle is 5° and the optical cut angle meets the PTP at 10.1°, the incident light entering the AOTF normally is diffracted in a central wavelength of 10.6 μm. When the external incident angle in the YOZ plane is −5°, the central wavelength of the diffracted light is 11.08 μm, and the spectral bandwidth is 1.3 times that of the normal incidence; when the external incident angle in the YOZ plane is 5°, the central wavelength is 10.87 μm, and the spectral bandwidth is 0.8 times that of the normal incidence. On the XOZ plane, when incident at an angle of 5° or −5°, the central wavelength of the diffracted light is 10.96 μm. Variations in the central wavelength and spectral bandwidth primarily occur in the YOZ plane.

For spectral imaging applications requiring high spectral finesse, it is necessary to increase the *L* and α. However, acoustic waves with larger acoustic angles have higher acoustic velocities within the AO crystal, leading to a decrease in the acousto-optic figure of merit, and consequently reducing the device’s diffraction efficiency. Therefore, for AOTFs that are used in imaging systems operating in high-intensity light conditions, a simultaneous increase in the acoustic angle and transducer length can be used in the AOTF design. For AOTFs that require a better diffraction efficiency and high throughput, a smaller α is often chosen, and *L* is enlarged as much as possible.

**Figure 4:**
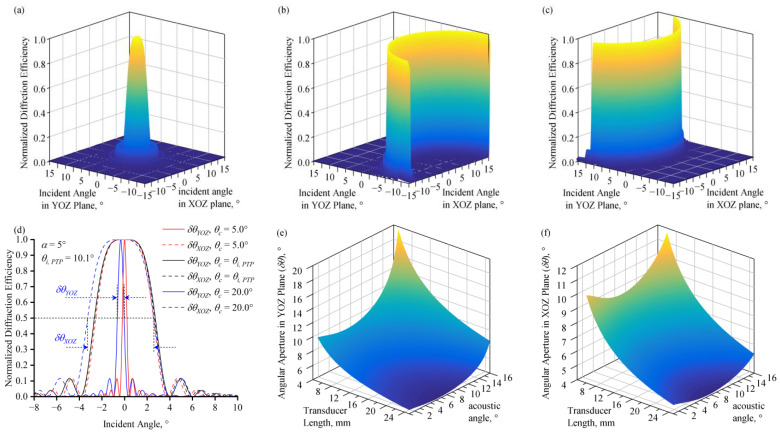
(**a**) The normalized diffraction efficiency (*α* = 5°, *θ_c_* = 10.1°, *L* = 15 mm). (**b**) The normalized diffraction efficiency (*α* = 5°, *θ_c_* = 20°, *L* = 15 mm). (**c**) The normalized diffraction efficiency (*α* = 5°, *θ_c_* = 5°, *L* = 15 mm). (**d**) The normalized diffraction efficiency in YOZ and XOZ planes. (**e**) The monochromatic angular aperture in the YOZ plane varies with acoustic angle and transducer length. (**f**) The monochromatic angular aperture in the XOZ plane varies with acoustic angle and transducer length.

The normalized diffraction efficiency at different incident angles of monochromatic light was calculated. When the optical cut angle, *θ_c_*, and the acoustic angle, *α,* satisfy the PTP, the light which is normally incident on the AOTF’s incident surface has an in-crystal incident angle that satisfies d*η*/d*θ_i_* = 0. Therefore, the normalized diffraction efficiency decreases from the peak at a slow rate with the external incident angle (Figure 4a). Consequently, AOTFs whose configuration satisfies the PTP obtain a larger monochromatic angular aperture. With an acoustic angle of 5° and a corresponding PTP incident angle of 10.1° at 10.6 μm, the black lines in Figure 4d represent the normalized diffraction efficiency of monochromatic light with variations in the external incident angles in the XOZ and YOZ planes. Two curves are close to overlapping. Large monochromatic angular apertures of δ*θ* = 5.4° are obtained in both planes.

When *θ_c_* and *α* do not satisfy the PTP, a large monochromatic angular aperture is not obtained, and the response distribution of monochromatic incident light at different angles exhibits various types [1]. For instance, when the acoustic angle is 5° and the optical cut angles are 5° and 20° respectively, the normalized diffraction efficiency across the entire field of view, as shown in Figure 4b,c, presents a non-rotationally symmetric form. The angular aperture in the YOZ plane, δ*θ_YOZ_*, significantly decreased to less than 1°.

The monochromatic angular apertures satisfying the PTP in two planes under different design parameters were calculated, as shown in Figure 4e,f. Both δ*θ_YOZ_* and δ*θ_XOZ_* decrease with the increase in transducer length. Both δ*θ_YOZ_* and δ*θ_XOZ_* first decrease slowly and then increase when *α* increases. When *L* = 15 mm, δ*θ_XOZ_* reaches its minimum at *α* = 7.5°, and δ*θ_YOZ_* at *α* = 6.25°, with both angular apertures having a minimum value of about 5.4°.

**Figure 5:**
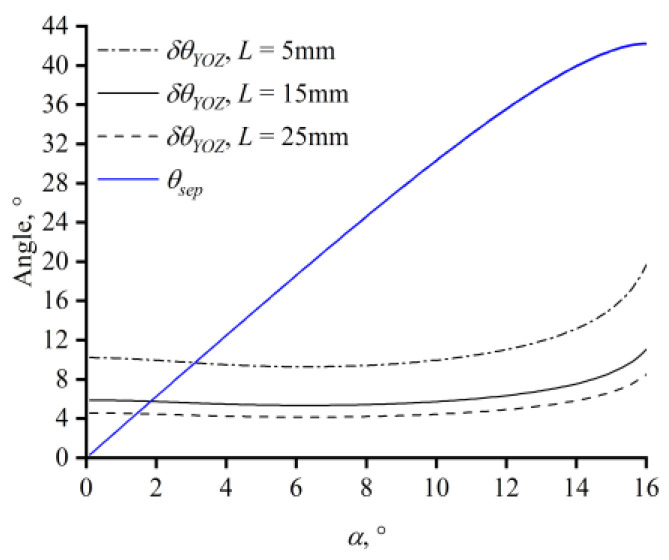
External separation angle and monochromatic angular aperture with different transducer lengths.

To ensure that the transmitted light does not overlap with the diffracted light, the aperture angle of the AOTF on the YOZ plane should not exceed the separation angle. For Hg_2_Br_2_ crystals, the separation angle monotonically increases with the acoustic angle (Figure 5) and exceeds the monochromatic angular aperture for transducer lengths of 15 mm and 5 mm at acoustic angles of 1.9° and 3.2°, respectively.

The optical throughput of the AOTF can be estimated based on the device’s angular aperture, spectral bandwidth, and diffraction efficiency. The transmission function *Φ* is shown below.

(8) $$\Phi (\lambda_{0},\lambda ,\alpha ,L,\delta \theta_{X},\delta \theta_{Y},\delta \lambda )=\int_{-\delta \theta_{X}}^{+\delta \theta_{X}}d\theta_{X}\int_{-\delta \theta_{Y}}^{+\delta \theta_{Y}}d\theta_{Y}\int_{\lambda_{0}-\delta \lambda /2}^{\lambda_{0}+\delta \lambda /2}\eta (\lambda ,\alpha ,L,l)d\lambda$$

Here, *λ_0_* and *δλ* are the central wavelength and the spectral bandwidth of the waveband. *θ_X_* and *θ_Y_* are the angular apertures of the AOTF in the XOZ and YOZ planes, respectively. ***l*** is a vector composed of the cosine of the direction of the incident light. Increasing the angular aperture and spectral bandwidth is beneficial for enhancing the optical throughput of the AOTF, thereby improving the light-gathering capability of the spectral imaging system. To increase the angular aperture, it is necessary to increase the acoustic angle or decrease the transducer length. To widen the spectral bandwidth and improve the diffraction efficiency, it is necessary to decrease the acoustic angle or increase the length of the transducer. By controlling the incident cut angle and the acoustic cut angle, a wide spectral bandwidth and large angular aperture can be achieved, endowing the device with the advantage of high throughput. However, this also leads to the drawback of a reduced spectral resolution. Moreover, a larger angular aperture also implies greater optical aberrations.

**Figure 6:**
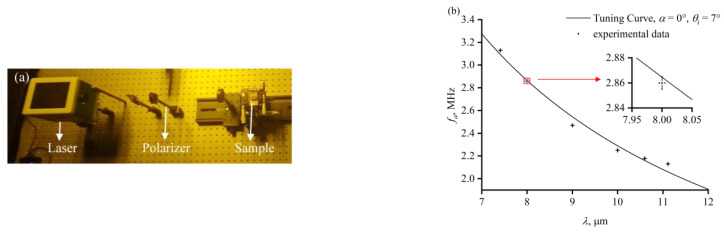
(**a**) The photograph of the experiment. (**b**) Theoretical tuning curve of the Hg_2_Br_2_ AOTF sample and the measured data.

An Hg_2_Br_2_-based AOTF sample was prepared to verify its diffraction capability in the long-wave infrared band. In the AO crystal of the sample, the acoustic angle is 0°, at which point the relationship between the incident angle of the optical wave and the phase-matching drive frequency is close to linear, which can facilitate experimental adjustments based on the relationship between the drive frequency, the angle of the light, and the light wavelength. The incident angle of the optical wave in the AO crystal is 7°. A long-wave infrared laser (Block Engineering, Southborough, MA, USA) was used as a light source in the experiment, as shown in Figure 6a. Laser beams with different wavelengths illuminated the incident surface of the AOTF. The frequency of the RF signal loaded on the piezoelectric transducer was adjusted until the diffracted light intensity measured by a power meter (Thorlabs, Newton, NJ, USA) was at its highest. The drive frequency was recorded with each wavelength of the laser beam. During the experiment, the experimental environment was room temperature. After the activation of the laser and the sample’s power supply, a few minutes of warm-up were needed to ensure that the laser power, the AOTF sample’s supply power, and the AO crystal’s temperature all remained stable. Then, the drive frequency was changed in increments of 0.01 MHz after recording the optical power. The measurement was repeated several times at each wavelength, and the measured values of the drive frequency corresponding to the peak diffraction showed good repeatability. The theoretical tuning curve of the sample and the measured data are shown in Figure 6.

In recent years, studies have been carried out on the spectral bandwidth and the Hg_2_Br_2_ AOTF. The variations in spectral bandwidth with light wavelength, incident angle, and acoustic angle were analyzed and experimentally verified in the YOZ plane, where the incident angle in the XOZ plane is zero [28]. A frequency bandwidth caused by phase mismatch has also been observed in the YOZ plane [32]. The concept of increasing the separation angle to enlarge the angular aperture of the AOTF to increase optical throughput was proposed [33]. An Hg_2_Br_2_ crystal device was constructed and its long-wave infrared diffraction ability was verified [25]. Compared to these studies, this paper calculates the spectral bandwidth of diffracted light in three-dimensional space, where the angle in the XOZ plane could be non-zero. To enhance the optical throughput of spectral imaging systems, improvements in the AOTF’s angular aperture and spectral bandwidth are considered. Thus, this study analyzes how AOTF performance parameters that affect optical throughput are influenced by design parameters.

## 4. Conclusions

In this investigation, we considered phase mismatches involving characteristic parameters, including spectral bandwidth and angular aperture, in the design of mercurous bromide crystal-based AOTF. The optical throughput of AOTF for spectral imaging applications can be improved by either broadening the spectral bandwidth or increasing the angular aperture. Both performance parameters depend on the device’s design parameters, which include the acoustic cut angle, optical cut angle, and the length of the piezoelectric transducer. A smaller acoustic cut angle and transducer length can yield a wider spectral bandwidth, but an excessively small acoustic cut angle also results in a too-small separation angle, limiting the AOTF’s angular aperture. The monochromatic angular aperture is most affected by the transducer length. A larger monochromatic angular aperture can be achieved with a shorter transducer length. Taking peak diffraction efficiency into consideration, an AOTF for high-throughput imaging systems should employ a smaller acoustic cut angle and as long a piezoelectric transducer as possible. A mercurous bromide AOTF sample was fabricated and tested. The obtained data verified the sample’s long-wave infrared diffraction ability. The research presented in this article can assist in the selection of the crystal configuration and transducer parameters of an AOTF at the design stage, which is of significant importance for achieving high-throughput AOTFs and spectral imaging systems.

## Data Availability

Data are contained within the article.

## References

[1] Gorevoy A.V., Machikhin A.S., Martynov G.N., Pozhar V.E. (2021). Spatiospectral transformation of noncollimated light beams diffracted by ultrasound in birefringent crystals. Photonics Res..

[2] Mantsevich S.N., Korablev O.I., Kalinnikov Y.K., Ivanov A.Y., Kiselev A.V. (2015). Wide-aperture TeO_2_ AOTF at low temperatures: Operation and survival. Ultrasonics.

[3] Korablev O.I., Belyaev D.A., Dobrolenskiy Y.S., Trokhimovskiy A.Y., Kalinnikov Y.K. (2018). Acousto-optic tunable filter spectrometers in space missions [Invited]. Appl. Opt..

[4] He Z., Li C., Xu R., Lv G., Yuan L., Wang J. (2019). Spectrometers based on acousto-optic tunable filters for in-situ lunar surface measurement. J. Appl. Remote Sens..

[5] Korablev O., Ivanov A., Fedorova A., Kalinnikov Y.K., Shapkin A., Mantsevich S., Viazovetsky N., Evdokimova N., Kiselev A.V., Scholl M.S., Paez G. (2015). Development of a mast or robotic arm-mounted infrared AOTF spectrometer for surface Moon and Mars probes. Infrared Remote Sensing and Instrumentation XXIII, Proceedings of the SPIE Optical Engineering + Applications, San Diego, CA, USA, 9–13 August 2015.

[6] Gupta N., Andresen B.F., Fulop G.F., Norton P.R. (2008). Hyperspectral imager development at Army Research Laboratory. Infrared Technology and Applications XXXIV, Pts 1 and 2, Proceedings of the SPIE Defense and Security Symposium, Orlando, Florida, United States, 16–20 March 2008.

[7] Machikhin A., Batshev V., Pozhar V., Naumov A., Gorevoy A. (2018). Acousto-optic tunable spectral filtration of stereoscopic images. Opt. Lett..

[8] Goutzoulis A.P., Pape D.R., Kulakov S.V. (1994). Design and Fabrication of Acousto-Optic Devices.

[9] Chang I.C. (1977). Tunable Acousto-Optic Filters: An Overview. Opt. Eng..

[10] Chang I.C. (1974). Noncollinear Acousto-Optic Filter with Large Angular Aperture. Appl. Phys. Lett..

[11] Yushkov K.B., Chizhikov A.I., Makarov O.Y., Molchanov V.Y. (2020). Optimization of Noncollinear AOTF Design for Laser Beam Shaping. Appl. Opt..

[12] Jensen J.O., Soos J., Qadri S., Jin F., Jensen J., Gupta N., Diestler M., Trivedi S., Kim J., Amarasinghe P. (2017). Long wavelength infrared (LWIR) AOTF and AOM modulators using Hg_2_Br_2_ crystals. Infrared Sensors, Devices, and Applications VII, Proceedings of the SPIE Optical Engineering + Applications, San Diego, CA, USA, 6–10 August 2017.

[13] Batshev V.I., Machikhin A.S., Kozlov A.B., Boritko S.V., Sharikova M.O., Karandin A.V., Pozhar V.E., Lomonov V.A. (2020). Tunable Acousto-Optic Filter for the 450-900 and 900-1700 nm Spectral Range. J. Commun. Technol. Electron..

[14] Dyakonov E., Porokhovnichenko D., Ryu J., Balakshy V. (2021). Implementation of the wide-angle acousto-optical interaction geometry in a mercury bromide single crystal. Appl. Opt..

[15] Yushkov K.B., Anikin S.P., Gurov V.V., Kolesnikov A.I., Molchanov V.Y., Tatarnikov A.M., Potanin S.A., Esipov V.F., Evans C.J., Simard L., Takami H. (2018). Acousto-optic spectrometer for speckle imaging. Ground-Based and Airborne Instrumentation for Astronomy VII.

[16] Gupta N., Voloshinov V.B. (2007). Development and characterization of two-transducer imaging acousto-optic tunable filters with extended tuning range. Appl. Opt..

[17] Kostyleva E.I., Mantsevich S.N. Quasicollinear Geometry of Acousto-Optical Interaction in Mercury Halide Crystals. Proceedings of the 2023 Wave Electronics and Its Application in Information and Telecommunication Systems (WECONF).

[18] Hurtado-Aviles E.A., Torres J.A., Trejo-Valdez M., Urriolagoitia-Sosa G., Villalpando I., Torres-Torres C. (2017). Acousto-Plasmonic Sensing Assisted by Nonlinear Optical Interactions in Bimetallic Au-Pt Nanoparticles. Micromachines.

[19] Krauz L., Pata P., Bednar J., Klima M. (2021). Quasi-collinear IR AOTF based on mercurous halide single crystals for spatio-spectral hyperspectral imaging. Opt. Express.

[20] Barta C., Barta C. (1990). Physical properties of single crystals of the calomel group (Hg_2_X_2_: X=Cl, Br). Mater. Sci. Forum.

[21] Bass M., Decusatis C., Enoch J. (2009). Handbook of Optics.

[22] Gupta N., Dhar N.K., Dutta A.K. (2014). Materials for Imaging Acousto-Optic Tunable Filters. Image Sensing Technologies: Materials, Devices, Systems, and Applications.

[23] Porokhovnichenko D., Ryu J., Zinkin D., Voloshinov V. Analysis of wide-angle acousto-optic interaction geometry in single crystal mercury bromide. Proceedings of the Fourteenth School on Acousto-Optics and Applications.

[24] Machikhin A., Batshev V., Pozhar V. (2017). Aberration analysis of AOTF-based spectral imaging systems. J. Opt. Soc. Am. A-Opt. Image Sci. Vis..

[25] Amarasinghe P.M., Kim J., Trivedi S., Jin F., Soos J., Diestler M., Qadri S.B., Jensen J.L., Jensen J., Gupta N. (2021). Mercurous Bromide (Hg_2_Br_2_) Acousto-Optic Tunable Filters (AOTFs) for the Long Wavelength Infrared (LWIR) Region. J. Electron. Mater..

[26] Zhang H., Zhao H., Guo Q., Xu D., Teng W. (2023). Polarization-Multiplexed High-Throughput AOTF-Based Spectral Imaging System. Materials.

[27] Voloshinov V.B., Bogomolov D.V. (2005). Acouso-optic processing of images in ultraviolet, visible and infrared regions of spectrum. Acousto-Optics and Photoacoustics, Proceedings of the Congress on Optics and Optoelectronics, Warsaw, Poland, 28 August–2 September 2005.

[28] Georgiev G., Glenar D.A., Hillman J.J. (2002). Spectral characterization of acousto-optic filters used in imaging spectroscopy. Appl. Opt..

[29] Zhao H., Cheng C., Guo Q., Yu K., Yang Y. (2024). Angular-Spectral Characteristics of Acousto-Optic Tunable Filters Based on Mercurous Halide Crystals. Materials.

[30] Valle S., Ward J., Pannell C., Johnson N.P. (2015). Acousto-optic tunable filter for imaging application with high performance in the IR region. Optical Components and Materials XII, Proceedings of the SPIE OPTO, San Francisco, CA, USA, 7–12 February 2015.

[31] Yu K., Guo Q., Zhao H., Cheng C. (2023). The Calibration Methods of Geometric Parameters of Crystal for Mid-Infrared Acousto-Optic Tunable Filter-Based Imaging Systems Design. Materials.

[32] Zhang H., Zhao H., Guo Q., Xuan Y. (2023). Calibration of Acousto-Optic Interaction Geometry Based on the Analysis of AOTF Angular Performance. Materials.

[33] Voloshinov V.B., Mosquera J.C. (2006). Wide-aperture acousto-optic interaction in birefringent crystals. Opt. Spectrosc..

